# Reliability and Validity of Ankle Muscle Strength Testing Using the ISOTIB Device

**DOI:** 10.1002/jfa2.70098

**Published:** 2026-04-09

**Authors:** Sean Drew, Sean A. Horan, Steven Duhig

**Affiliations:** ^1^ School of Allied Health, Sport and Social Work Griffith University Gold Coast Australia

**Keywords:** electromyography, foot and ankle, instability, muscle strength dynamometer, resistance training

## Abstract

**Introduction:**

One repetition maximum (1RM) testing is recognised as a reliable and valid method for determining maximum muscle strength. However, there are limited reports in the literature for measuring maximum ankle strength using free‐weight methods. The aim of this study was to determine the test‐retest reliability and concurrent validity of a novel free‐weight device (ISOTIB) used to measure ankle strength.

**Methods:**

Fifteen healthy, recreationally active, adults (male = 10, female = 5, age = 29.7 ± 4.4 years) volunteered for the study, attending two sessions 1 week apart. Reliability was assessed using intraclass correlation coefficients (ICC_(3,1)_) and Bland‐Altman method. Concurrent validity was examined by comparing 1RM ankle strength and normalised muscle activity using surface electromyography (sEMG) during dorsiflexion, inversion, and eversion movements performed with the ISOTIB device and an isokinetic dynamometer.

**Results:**

The ISOTIB exhibited excellent test‐retest reliability for maximal dorsiflexion (ICC_(3,1)_ = 0.99, 95% CI: 0.977–0.997), inversion (ICC_(3,1)_ = 0.99, 95% CI: 0.970–0.997), and eversion (ICC_(3,1)_ = 0.97, 95% CI: 0.920–0.991) strength. Concurrent validity was confirmed, with high positive correlations for maximal dorsiflexion (*r* = 0.90, 95% CI: 0.72–0.97) *p* < 0.001), inversion (*r* = 0.67, 95% CI: 0.24–0.88) *p* < 0.007), and eversion (*r* = 0.87, 95% CI: 0.64–0.96) *p* < 0.001) strength. sEMG results supported concurrent validity with comparable activity in the tibialis anterior, peroneus longus, and peroneus brevis muscles.

**Conclusion:**

The ISOTIB demonstrated excellent reliability and validity, suggesting it is a viable tool for assessing maximal ankle strength. The ISOTIB offers a practical alternative to current methods, which are typically expensive and utilise large immovable devices.

## 1 Introduction

The one repetition maximum (1RM) strength test is required to determine an individual's maximum liftable weight for a particular exercise or movement task [1, 2, 3, 4]. These tests often use exercises such as the bench press or back squat to measure an individual's upper or lower body's force generating capacity. The reliability and validity of these 1RM strength testing protocols is well established in the literature [5, 6, 7, 8, 9]. Results from 1RM testing are used to determine appropriate training loads for individuals by calculating the relative intensity of exercise loads and tailoring load prescription to ensure adequate training stimulus and progressive overload [10]. Adopting a structured and tailored resistance training approach using specific variables of load, volume, rest, and tempo enables individuals to target and optimise different training outcomes such as endurance, hypertrophy, maximal strength, and power [11]. The gold standard for single joint muscle strength assessment is isokinetic dynamometry [12, 13]. Isokinetic dynamometry measures torque while controlling for factors such as speed (e.g., slow, fast) and contraction type (e.g., isometric, eccentric, concentric). Assessments generally involve single joint contractions, where an individual moves through a given range of movement (e.g., extension and flexion of the knee joint). This approach measures torque throughout the available range of motion and in each direction of movement, as permitted by the dynamometer. Such methods have been valuable in optimising rehabilitation programmes following recovery from injury [14, 15]. However, despite these benefits, isokinetic dynamometers have limitations. During an isokinetic‐based strength test, the resistance of the device adapts to the subject's output while the speed of movement is held constant. Consequently, the natural variation in force and cadence seen with free weights is not evident, which may reduce physiological validity. Another major limitation of isokinetic dynamometers is their inaccessibility owing to their large size, cost, and lack of portability. These limitations highlight the need for valid field‐based strength measurement tools [16].

Although established protocols exist for assessing strength of the major muscle groups of the upper and lower limb using an isokinetic dynamometer [15, 17], there is a need for methods to assess maximal ankle strength using free‐weights to overcome the highlighted barriers. The recently developed ISOTIB (HGG Performance, QLD, Australia) is a portable foot‐mounted resistance training device designed for unilateral loading to isolate and strengthen the lower limb muscles responsible for ankle dorsiflexion (tibialis anterior), inversion (tibialis anterior and posterior), and eversion (peroneus longus, peroneus brevis). The ISOTIB is an open chain exercise device where the foot limb moves freely in space and is not fixed to a surface. It features a durable nylon frame with a stainless steel 50 mm loading bar compatible with Olympic plates, allowing for progressive overload. The loading bar can be positioned in three configurations to adjust weight distribution based on foot size and exercise type. An adjustable ratchet strap system with five strap settings and a rear heel hoop ensures a secure fit during movement. While initially developed as an exercise training tool, it has the potential to be used as a practical method for assessing 1RM ankle strength given load can be changed easily, and its portability and low cost. Progressive muscle overload is a well‐established principle of strength training used to maximise training benefits [18, 19, 20, 21] and a major advantage when using the ISOTIB compared with other strength based modalities such as resistance bands [22], as it allows for precise load adjustments and significantly greater resistance, similar to weight training equipment. Therefore, the aim of this study was to evaluate the ISOTIB as an ankle strength assessment tool by establishing its test‐retest reliability and concurrent validity with the gold standard, isokinetic dynamometry.

## 2 Methods

A repeated measures design was used to determine the test‐retest reliability of the ISTOTIB 1RM ankle strength test. Test‐retest reliability was assessed by comparing 1RM testing results obtained at two different testing sessions, separated by 7 days. A period of 7 days allowed participants to recover from any muscle soreness associated with 1RM testing, but to limit any adaptations to normal training routines. Concurrent validity was evaluated by comparing the weight lifted using the ISOTIB and the peak torque outputs during the same ankle movements using an isokinetic dynamometer (HUMAC NORM, Stoughton, MA, USA). Muscle activation was assessed using surface electromyography (sEMG), by comparing muscle activity of three separate muscles during the ISOTIB 1RM test and isokinetic dynamometer maximum voluntary contraction (MVC) test.

### 2.1 Participants

Fifteen healthy, recreationally active, males and females volunteered to participate in the study with demographic information presented in Table 1. To be eligible, participants had to be moderately active and exercising an average of three times a week for an hour at moderate intensity over the previous 6 months. Participants with a history of orthopaedic surgery or musculoskeletal injuries in the lower leg region within the past 6 months were excluded. Before commencing, all participants received a written description of the study, including potential risks, and provided informed consent. Ethical approval was granted by the Griffith University Ethics Review Committee (Ref No: 2023/171). Sample size was estimated based on the method described by Borg [23] for reliability studies using the intraclass correlation coefficient (ICC_(3,1)_). Using a target ICC_(3,1)_ of 0.95 (excellent reliability) [24], a lower confidence limit of 0.75, alpha = 0.05, 80% statistical power, and two repeated measurements per participant, a minimum of 12 participants was determined.

**TABLE 1:** Participant characteristics (mean ± standard deviation).

Characteristic	Men (*n* = 10)	Women (*n* = 5)	Combined (*n* = 15)
Age (years)	31.7 ± 3.7	25.8 ± 2.8	29.7 ± 4.4
Height (cm)	181.8 ± 4.2	164.6 ± 2.9	176.1 ± 9.2
Weight (kg)	84.9 ± 7.9	64.9 ± 10.9	78.2 ± 13.0

### 2.2 Surface Electromyography

Muscle activity of the tibialis anterior, peroneus longus, and peroneus brevis was recorded during all ankle strength tests using sEMG (Cometa MiniWave Wireless EMG System, Milan, Italy). Electrode placement areas were shaved, abraded, and cleaned with alcohol. sEMG data were collected by following a standardised bipolar electrode placement procedure using SENIAM guidelines [25]. All sEMG data were sampled at 2000 Hz and collected during the second testing session.

The sEMG recordings were standardised by first normalising to maximum voluntary isometric contractions (MVIC). Peak sEMG activation levels were extracted from EMG linear envelopes for data analysis. All sEMG data were processed using Matlab software (Matlab_R2023a, Mathworks Inc., Natick, MA, USA) using MOtoNMS [26] whereby the signals were detrended (remove direct current offset), bandpass filtered (second order, 30–300 Hz), full‐wave rectified, low pass filtered (second order, 6 Hz) using zero‐lag Butterworth filters [27, 28]. sEMG signals were then normalised to the maximal EMG value identified within each strength test across all tasks of the dynamic movements and maximum voluntary isometric contractions (MVICs), resulting in normalised linear envelopes [29, 30]. Specifically, each participants own sEMG data from ISOTIB test were normalised to the maximum value observed across 1RM ISOTIB trials and MVICs while sEMG data from the isokinetic dynamometer were normalised to the maximum value observed across dynamometer MVC trials and MVICs. This approach ensured each device's sEMG results were independently normalised to the highest muscle activity obtained within that testing modality.

### 2.3 Testing Session 1

**FIGURE 1:**
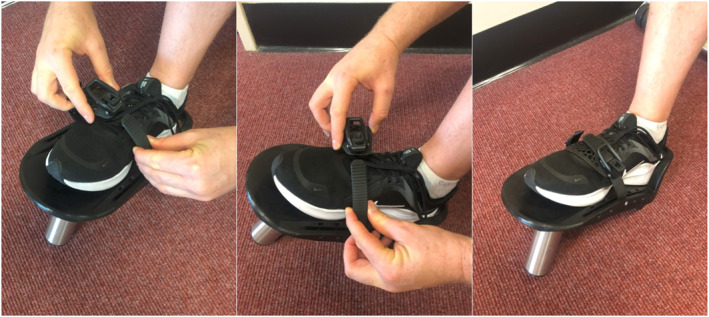
The ISOTIB free‐weight device and foot positioning utilised during 1RM strength testing.

**FIGURE 2:**
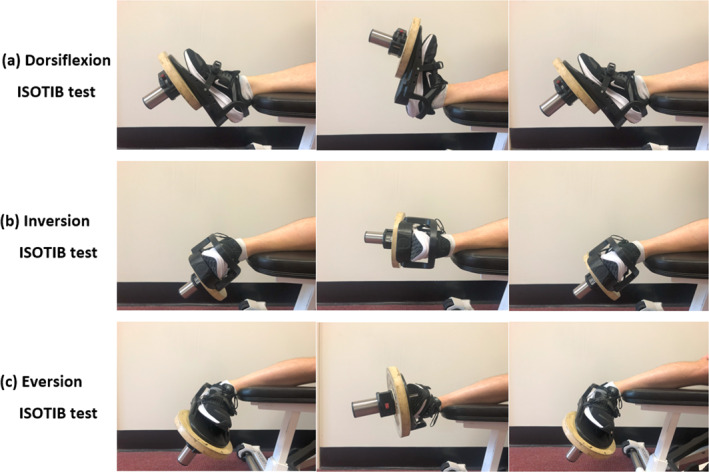
Participant positioning used to assess ISOTIB 1RM strength for ankle (a) dorsiflexion, (b) inversion, and (c) eversion.

Participants were familiarised with both the ISOTIB and dynamometer devices before testing. Maximal concentric strength assessments were performed for dorsiflexion, inversion, and eversion through the participant's available range of motion. ISOTIB testing began with the participant seated on a weight bench, foot on the ground and were instructed to use their dominant leg for testing. The ISOTIB foot‐plate was attached to the foot using a heel lock and ratchet strap (Figure 1). Participants wore sports shoes for all tests. An estimated submaximal load (∼60% of perceived maximal effort) for warm‐up was applied to the ISOTIB and secured with a barbell clip. Participants were assisted into position: seated with the ankle joint aligned with the bench edge (Figure 2). For inversion and eversion tests, foot orientation was adjusted so the lateral or medial foot faced upwards.

Participants performed four dorsiflexion reps, followed by 60 s rest. If the load was inappropriate, it was adjusted, and the set repeated. Next, participants performed three reps at a higher load (∼80% of perceived maximal effort), followed by 2 min rest. Then 1RM attempts commenced. If two repetitions were completed, the load was increased until only one rep could be performed. Form was monitored by ensuring consistent leg and foot orientation and maintaining a fixed knee angle throughout the movement. Two minutes rest were given between attempts. 1RM was typically achieved within two to four attempts. After dorsiflexion testing, participants rested for 5 min before inversion testing, then 5 min rest before eversion testing.

For isokinetic dynamometry testing, participants sat with the dominant test leg supported under the thigh at 90° hip/knee flexion. The leg and foot were secured with Velcro straps. Strength assessments for dorsiflexion, inversion, and eversion were conducted at 60°/s through full range of motion.

Isokinetic MVCs were collected at 60°/s, a slow–moderate velocity that maximises peak torque expression while minimising errors caused by momentum, consistent with the force–velocity relationship [31]. This speed is commonly adopted for ankle and lower‐limb MVC reliability testing, providing high reproducibility [24]. Testing began with two submaximal sets as warm‐up of three dorsiflexion repetitions at ∼60% of perceived maximal effort, followed by 60 s rest, then three repetitions at ∼80% of perceived maximal effort. After a two‐minute rest, participants performed two trials of three maximum effort repetitions or MVCs. After dorsiflexion‐plantarflexion testing, participants rested for 5 min before inversion‐eversion testing. Peak torque (Nm) from maximal contractions was used for analysis. This selected dynamometer protocol [32] aligns with our objective of assessing lower limb strength and ensures participants were prepared to perform maximal effort without being fatigued. A minimal warm up (two submaximal sets) were included given the 1RM ISOTIB test was completed earlier in the same session. Two MVC trials were performed to properly achieve the highest result if the first attempt was substandard and two attempts are typically given for reliability studies using an isokinetic dynamometer [24].

### 2.4 Testing Session 2

Testing session two was conducted 7 days after session one. sEMG data were collected during all ISOTIB and dynamometer strength assessments for this session. Since participants had already been familiarised in Session 1, they were accustomed to the testing procedures, which allowed for more consistent execution and higher‐quality EMG recordings. Session two began with three MVIC trials (5‐s holds) using the footplate in a locked setting for dorsiflexion, inversion, and eversion using the same dynamometer setup as session one, with two‐minute rest intervals. MVIC trials were used to normalise sEMG data. After MVIC collection, participants completed ISOTIB and dynamometer maximal strength assessments following the same procedures as session one.

### 2.5 Statistical Analysis

Normal distribution was evaluated using Shapiro‐Wilk testing in SPSS (Version 29, Chicago, IL). Descriptive statistics are presented as mean ± standard deviation (SD) and Cohen's *d* effect sizes (ES). An effect size < 0.20 was considered trivial, 0.20–0.49 small, 0.50–0.79 moderate, and ≥ 0.80 large [18]. Intraclass correlation coefficients (ICCs_(3,1)_) with 95% confidence intervals (CIs) were calculated using a two‐way mixed‐effects model, single measurement, absolute agreement, which is appropriate for assessing test‐retest reliability when the same measurement device and procedure are used across all participants to compare reliability between maximal ISOTIB strength tests. Reliability was interpreted using the lower bound of the 95% CI [33]. ICC_(3,1)_ values < 0.5 were considered poor, 0.5–0.75 moderate, 0.75–0.9 good, and > 0.9 excellent [5]. Test‐retest reliability was examined using Bland‐Altman plots, calculating 95% limits of agreement and mean bias between test sessions. To assess validity, Pearson's correlations coefficient compared 1RM ISOTIB and dynamometer MVC tests. Correlation strength was defined a priori: *r* = 0.2–0.39 (low), 0.4–0.59 (moderate), 0.6–0.79 (high), and 0.8–1.0 (very high) [17]. Corresponding 95% confidence intervals (CIs) were calculated using the Fisher *Z* transformation method in Excel [34]. Statistical analysis of sEMG data assessed significant differences in peak activation between ISOTIB and dynamometer tests. Data with normal distribution were analysed with paired sample t‐tests; non‐normal data were analysed using the Wilcoxon Signed‐Rank Test. Outliers (> 3 SD from mean) were removed prior to conducting data analysis [16].

## 3 Results

### 3.1 Reliability

**FIGURE 3:**
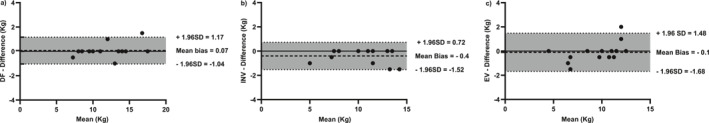
Bland‐Altman plots illustrating mean bias (dashed line) and limits of agreements (upper and lower dotted lines) for test‐retest reliability for 1RM ankle strength testing using the ISOTIB device (*n* = 15); (a) dorsiflexion, (b) inversion, and (c) eversion. DF, dorsiflexion; EV, eversion; INV, inversion; kg, kilogram.

The ICC_(3,1)_ values for the ISOTIB and dynamometer are presented in Table 2. Using the lower bound of the 95% CI, ICCs_(3,1)_ ranged from 0.97 to 0.99 across all ankle movements, indicating excellent agreement. Bland–Altman plots (Figure 3) revealed a mean difference of 0.07 kg for dorsiflexion, −0.4 kg for inversion, and −0.1 kg for eversion for the ISOTIB tests between sessions.

**TABLE 2:** Test‐retest reliability for ankle strength test measurements, for the 1RM ISOTIB test and maximum voluntary contraction dynamometer test.

Ankle strength test	Session 1 (*n* = 15)	Session 2 (*n* = 15)	ICC (CI 95%)
Dorsiflexion			
ISOTIB (kg)	11.6 ± 3.3	11.6 ± 3.1	0.99 (0.97–0.99)
Dynamometer (Nm)	25.5 ± 5.8	26.6 ± 5.9	0.95 (0.89–0.98)
Inversion			
ISOTIB (kg)	10.2 ± 2.9	10.6 ± 2.9	0.99 (0.97–0.99)
Dynamometer (Nm)	29.9 ± 11.9	31.4 ± 12.3	0.98 (0.94–0.99)
Eversion			
ISOTIB (kg)	9.6 ± 2.7	9.7 ± 2.3	0.97 (0.92–0.99)
Dynamometer (Nm)	21.1 ± 6.1	20.8 ± 6.5	0.98 (0.93–0.99)

*Note:* Data for session 1 and 2 are presented as mean ± standard deviation.

Abbreviations: CI, confidence interval; ICC, intraclass correlation coefficient; kg, kilogram; Nm, Newton‐metre.

### 3.2 Validity

**FIGURE 4:**
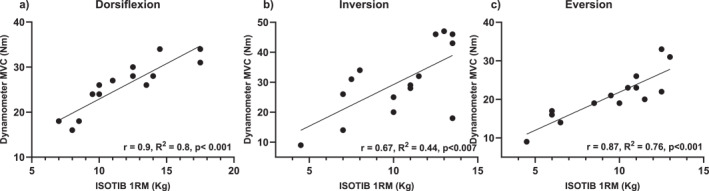
Relationship between 1RM ankle strength measured using the ISOTIB device and maximal isokinetic ankle strength (60°/s) measured using a dynamometer; (a) dorsiflexion, (b) inversion, and (c) eversion. 1RM, 1 Repetition maximum; kg, kilogram; MVC, maximal voluntary contraction; Nm: Newton‐metre.

Results for the 1RM ISOTIB strength tests and the isokinetic dynamometer tests demonstrated very high positive correlations for both the dorsiflexion (*r* = 0.9, 95% CI: 0.72–0.97, *p* < 0.001) and eversion (*r* = 0.87, 95% CI: 0.65–0.96, *p* < 0.001) directions, and a high positive correlation for the inversion (*r* = 0.67, 95% CI: 0.24–0.88, *p* < 0.007) direction (Figure 4).

**FIGURE 5:**
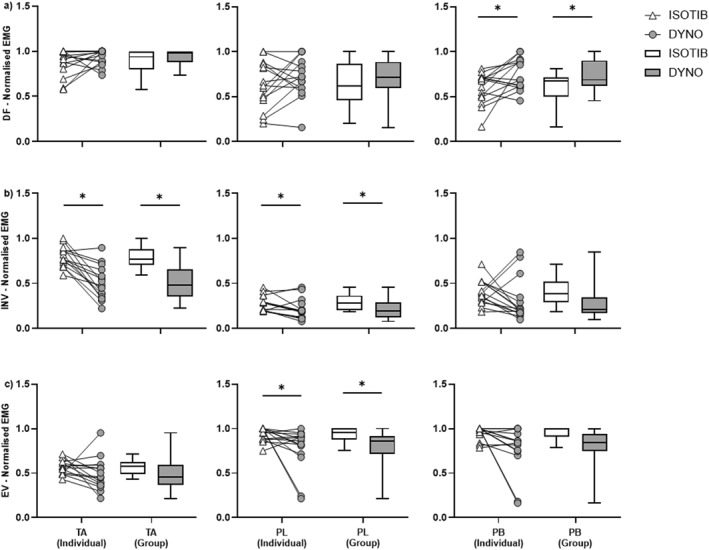
Normalised peak sEMG amplitude for tibialis anterior (TA), peroneus longus (PL) and peroneus brevis (PB) muscles during the ISOTIB 1RM test and the isokinetic dynamometer (DYNO) maximum voluntary contraction test for each ankle movement; (a) dorsiflexion, (b) inversion, and (c) eversion. Both individual (triangle and circle symbols) and group (white and grey box plots) sEMG amplitude data are presented. *Significant difference (*p* < 0.05) in peak sEMG amplitude between the ISOTIB and dynamometer strength tests.

Normalised sEMG amplitudes (0.0–1.0) for the tibialis anterior, peroneus longus, and peroneus brevis during 1RM ISOTIB and isokinetic strength testing across all movement directions are presented in Figure 5 as individual and group electromyography data. For dorsiflexion, peroneus brevis activity was lower during the ISOTIB test (59.4% ± 17.4) compared with the dynamometer test (75.3% ± 17.4; *p* = 0.0165, ES: 0.70). For inversion, tibialis anterior activity was higher during the ISOTIB test (79.9% ± 11.5) compared with the dynamometer test (50.5% ± 18.6; *p* = 0.00, ES: 1.9), and peroneus longus activity was also higher during the ISOTIB test (27.3% ± 8.9) compared with the dynamometer test (21.7% ± 11.8; *p* = 0.04, ES: 0.57).

For eversion, peroneus longus activity was higher during the ISOTIB test (94.0% ± 8.0) compared with the dynamometer test (78.0% ± 24.0; *p* ≤ 0.0479, ES: 6.33), and peroneus brevis activity was also higher during the ISOTIB test (95.0% ± 8.0) compared with the dynamometer test (77.0% ± 27.0; *p* ≤ 0.0126, ES: 1.34). All other sEMG results were not significantly different between the ISOTIB and dynamometer tests (see Figure 5). A total of three outliers were identified and excluded from sEMG data.

## 4 Discussion

This study is the first to assess the test‐retest reliability and concurrent validity of a novel open chain free weight strength training device for measuring ankle dorsiflexion, inversion, and eversion 1RM strength. The ISOTIB demonstrated excellent reliability and validity achieving comparable muscle activity to a gold standard dynamometer test verifying its use as a utility method for assessing ankle strength. Furthermore, the ISOTIB could offer a convenient and practical ankle strength training alternative to commonly used methods such as resistance bands and cable devices.

### 4.1 Test‐Retest Reliability

We examined test‐retest reliability of the 1RM ISOTIB ankle strength test for dorsiflexion, inversion, and eversion by comparing results from two testing sessions separated by 1 week. Intra‐class correlation results revealed that the ISOTIB demonstrated excellent reliability in all three directions of movement for which ankle strength was tested.

In line with Koo and Li [33], we interpreted reliability based on the lower bound of the 95% CI, a more conservative assessment. However, all lower bound ICC_(3,1)_ values remained within excellent range, confirming the ISOTIB's high reliability for maximal ankle dorsiflexion, inversion, and eversion strength testing. A recent paper [35] that examined the reliability of a 1RM ankle strength test using a cable‐pull machine reported lower levels of test‐retest reliability than our ISOTIB strength test. The cable‐pull machine ankle strength test demonstrated good reliability with ICCs_(3,1)_ of 0.88, 0.83, and 0.76 for ankle dorsiflexion, inversion, and eversion, respectively. The differences in reliability between the ISOTIB and cable‐pull machine test along with similar strength testing research [35] is likely due in part to differences in participant setup and positioning. During the cable‐pull machine ankle strength test, participants lay on the floor with their test leg positioned on a block and foot placed in a strap attached to a cable while a person manually held the participant’s leg in place. In such a position it would be possible for the participant’s leg to move, and also for the pressure placed on the leg to be uncomfortable which might affect the participant’s motivation. The cable‐pulley system could also provide varying levels of internal resistance depending on positioning and line of pull of the cable. These factors have the potential to affect the outcome for each cable‐pulley test and lead to the reduced reliability compared with the ISOTIB test. In our study, we measured strength of the dominant leg which is known to improve test‐retest reliability of lower limb strength tests, particularly for ankle dorsiflexion, inversion, and eversion [24], potentially contributing to the excellent reliability for the ISOTIB tests.

Other methods for assessing ankle strength reported include handheld dynamometery (HHD). Spink and colleagues [15] examined the reliability of measuring ankle dorsiflexion, inversion, and eversion isometric strength using a HHD, which was placed over the participant’s mid‐foot while the lower limb was stabilised proximal to the ankle joint by the tester. Good to excellent between session (1 week) reliability was reported with ICCs_(3,1)_ between 0.78 and 0.94 [36]. Interestingly, ankle eversion strength testing with a HHD was the least reliable method compared to dorsiflexion and inversion testing. This pattern was observed for the ISOTIB reliability results in our study, with the eversion strength test demonstrating the lowest average ICC_(3,1)_ values and widest 95% confidence intervals compared with the dorsiflexion and inversion tests. Ankle eversion and to an extent inversion, are more complicated movement patterns than dorsiflexion as they involve movement contributions from multiple joints and rely on greater synergistic control between multiple muscles compared with dorsiflexion [37]. This likely leads to greater variability when measuring ankle eversion strength, and is why reliability values in our study and that of others could be lower in the eversion direction of movement. A limitation when using HHD to measure ankle strength, is that maximum isometric strength is tested. The ISOTIB device requires individuals to move an external load through their entire range of ankle movement, which is more representative of how people typically move their ankle and more closely mimics exercises in strength training programmes.

In addition to excellent ICC_(3,1)_ results for the ISOTIB device, our Bland‐Altman analysis demonstrated small differences in strength test results between sessions for the ISOTIB strength tests. The mean bias for ankle dorsiflexion, inversion, and eversion were all less than ± 0.5 kg, indicating the ISOTIB tests did not tend to over‐ or under‐estimate ankle strength between sessions. We also detected modest limits of agreements of −1.68 to 1.48 kg across all three ankle strength tests (dorsiflexion, inversion, eversion) suggesting the ISOTIB demonstrated an acceptable range of variance that was not clinically significant. While limited reports of other ankle strength tests are available in the literature, our findings confirm the ISOTIB is more reliable than other commonly utilised ankle strength tests such as HHDs [38, 39]. In addition to excellent between session reliability, the ISOTIB ankle strength tests are simple, practical, and offer the advantage of being able to examine strength through an individual's full range of motion rather than isometrically. From a practical perspective, we recommend anyone considering using our 1RM testing protocol provides individuals with adequate familiarisation and monitors leg position particularly during eversion 1RM testing given the slightly lower levels of reliability for the ISOTIB eversion test.

### 4.2 Validity

Maximal strength assessments using isokinetic dynamometry have been shown to be reliable and valid for assessing concentric and eccentric strength of different upper and lower limb joints including the ankle [40, 41]. In our study, we compared maximal ankle strength testing using an isokinetic dynamometer with the ISOTIB device. Other studies using handheld dynamometry have adopted a similar approach to our study, although their strength tests have utilised isometric or ‘holding’ tests [42, 43, 44]. In our study, we observed a strong positive relationship for ankle dorsiflexion and eversion strength tests between the dynamometer and ISOTIB, and a moderate relationship between devices for the inversion strength test. For each direction of movement, the ankle dorsiflexion test had the strongest relationship (*r* = 0.90; *R* ^2^ = 0.80) which is likely due to several reasons. The dorsiflexion test position involved a seated position with the foot off the edge of a weight bench and was a more familiar position than inversion and eversion test positions which involved an ‘awkward’ side‐sitting position. This difference in positioning likely meant participants were more comfortable and able to exert more consistent performances during the dorsiflexion test, which is a factor during strength testing [44, 45]. Secondly, compared with dorsiflexion, performing open chain inversion and eversion movements of the ankle are relatively uncommon and considered more difficult than dorsiflexion [46, 47]. Inversion and eversion more commonly occur as a closed chain movement, particularly during the stance phase of walking and running. These differences likely caused greater variability during the inversion and eversion strength tests compared with the dorsiflexion tests, leading to a weaker relationship between the ISOTIB and dynamometer results for the inversion and eversion tests.

We examined concurrent validity by measuring EMG activity of the tibialis anterior, peroneus longus, and peroneus brevis muscles during the ISOTIB and dynamometer ankle strength tests. Analysis of EMG data demonstrated that for the ISOTIB 1RM tests (dorsiflexion, inversion, and eversion), participants achieved comparable and greater muscle activity than the corresponding dynamometer tests. EMG analysis also reveals the prime mover muscles demonstrated greater EMG activity during the ISOTIB version of each ankle strength test, compared with the dynamometer. While there was no difference in tibialis anterior activity during the ISOTIB and dynamometer dorsiflexion tests, there was on average 30% greater activity in the tibialis anterior during the ISOTIB inversion test, and on average 16% and 18% greater activity in the peroneus longus and brevis muscles during the ISOTIB eversion test. This pattern of increased muscle activity in the prime movers during the ISOTIB ankle strength tests verifies the device effectively targeted the key muscles during each of the three strength tests. It is feasible that greater EMG activity corresponds to a greater number of motor units being recruited during the ISOTIB tests, which could lead to increased muscle force outputs [48, 49]. This higher level of EMG activity could be advantageous from a strength training perspective if using the ISOTIB as a training or rehabilitation tool. Compared with resistance‐based machines and bands, greater coordination, stabilisation, and synergistic muscle control is required when using free‐weight devices such as the ISOTIB [50, 51]. Further research is required to build on our findings and determine if training with the ISOTIB alters muscle control strategies around the ankle during everyday activities such as walking, running, and jumping tasks.

## 5 Limitations

This study is not without limitations that warrant acknowledgement. Participants were required to perform many ankle movements during each test session. This included familiarisation and warm up trials, the 1RM attempts, and different movement directions performed on each device. While standardised rest periods were given and no participants reported feeling fatigued, it is possible some participants may have experienced some physical or mental fatigue during testing which could have affected test results. Familiarisation procedures for both the ISOTIB and dynamometer were purposely brief to limit testing time and not induce any fatigue. While this was achieved, it is possible some participants were not familiar with the use of each device which may have influenced the results obtained during testing. Our participant cohort was healthy and free from injury; therefore, our results may not apply to other populations. Only the dominant leg was assessed in this study, which may have improved ICC_(3,1)_ values due to greater neuromuscular familiarity. However, this limits the generalisability of the findings, as potential strength or coordination asymmetries between limbs were not accounted for.

### 5.1 Practical Applications

The ISOTIB is a reliable and valid tool for assessing 1RM ankle strength, offering a practical alternative to more cumbersome and expensive equipment. Its portability and ease of use enhances its utility across different settings, such as sports training facilities and clinical environments. Given ISOTIBs effectiveness in measuring 1RM ankle strength, the ISOTIB has the potential to be a valuable tool for determining the most effective load for strength‐based training and rehabilitation programmes targeting the ankle. Ensuring programmes are progressive is vital in ensuring adaptation, and this can only be achieved if an individual's maximum is known so that different sub‐maximal loads can be incorporated into their training or rehabilitation programme. From a practical perspective, we recommend anyone considering using our 1RM testing protocol is to provide individuals with adequate familiarisation and properly monitor leg position, particularly during eversion 1RM testing given the slightly lower levels of reliability for the ISOTIB eversion test. In terms of sports medicine, it is likely the ISOTIB would be a useful tool in the assessment and rehabilitation of people following a lower limb injury, and this should therefore be taken into consideration when using the ISOTIB.

## Author Contributions

**Sean Drew:** conceptualization (lead), methodology (lead), investigation (lead), software (lead), data curation (lead), formal analysis (lead), visualization (lead), writing – original draft (lead), and writing – review and editing (equal). **Sean A. Horan:** supervision (lead), methodology (supporting) validation (equal), and writing – review and editing (equal). **Steven Duhig:** supervision (lead), methodology (supporting), validation (equal), and writing – review and editing (equal).

## Funding

The authors have nothing to report.

## Conflicts of Interest

The authors declare no conflicts of interest.

## Data Availability

The data that support the findings of this study are available from the corresponding authors upon reasonable request.
